# The Sleep-Promoting Ventrolateral Preoptic Nucleus: What Have We Learned over the Past 25 Years?

**DOI:** 10.3390/ijms23062905

**Published:** 2022-03-08

**Authors:** Elda Arrigoni, Patrick M. Fuller

**Affiliations:** 1 Department of Neurology, Beth Israel Deaconess Medical Center, Harvard Medical School, Boston, MA 02215, USA; 2 Department of Neurological Surgery, University of California, Davis School of Medicine, Davis, CA 95618, USA

**Keywords:** arousal, EEG, insomnia, VLPO, thermoregulation, preoptic, hypothalamus

## Abstract

For over a century, the role of the preoptic hypothalamus and adjacent basal forebrain in sleep–wake regulation has been recognized. However, for years, the identity and location of sleep- and wake-promoting neurons in this region remained largely unresolved. Twenty-five years ago, Saper and colleagues uncovered a small collection of sleep-active neurons in the ventrolateral preoptic nucleus (VLPO) of the preoptic hypothalamus, and since this seminal discovery the VLPO has been intensively investigated by labs around the world, including our own. Herein, we first review the history of the preoptic area, with an emphasis on the VLPO in sleep–wake control. We then attempt to synthesize our current understanding of the circuit, cellular and synaptic bases by which the VLPO both regulates and is itself regulated, in order to exert a powerful control over behavioral state, as well as examining data suggesting an involvement of the VLPO in other physiological processes.

## 1. The Hypothalamus and Sleep: A Brief History

During the epidemics of encephalitis lethargica, which both preceded and followed the 1918 influenza pandemic, the Austrian neurologist Baron Constantin von Economo undertook a series of clinicopathologic studies on individuals who were rendered insomniac by this previously unrecognized type of encephalitis [1,2]. What he discovered during his post mortem analysis of the brains of these insomniac individuals was the presence of inflammatory lesions involving the anterior hypothalamus and, to varying degrees, the adjacent basal ganglia, the latter of which would explain the chorea that was often associated with insomnia. Based upon these findings, von Economo hypothesized that the anterior hypothalamic region comprised a “*schlafsteuerungszentrum*” or “sleep center”. He further hypothesized that this sleep center actively inhibited the cerebral cortex and thalamus to produce, in his words, “brain sleep”. Less than a decade later, Hess reported that he could produce sleep in cats by stimulating various regions of the hypothalamus and midbrain [3], although his findings stood at variance with those of Ranson and colleagues [4,5,6], who were never able to produce sleep in cats or monkeys following scattered lesions of the hypothalamus. In 1946, Nauta undertook an extensive and detailed study of the role of the hypothalamus in the regulation of sleep, in part an attempt to reconcile the findings of von Economo, Ranson and Hess [7]. None of Nauta’s control lesions, which included incisions dorsal to the ventral thalamus and unilateral cuts to the hypothalamus, resulted in insomnia, nor did they result in marked changes in the sleep–wake cycle. Nauta then employed a parapharyngeal approach, along with a “tour de maître”, a small hook-shaped knife, to mechanically and systematically inflict “transverse” lesions in the rat hypothalamus. Nauta found that his “lesions”, when situated in the caudal hypothalamus, tended to produce mild-to-extreme sleepiness, whereas lesions encompassing the rostral lateral hypothalamus produced a state of insomnia. From this, Nauta concluded that the rostral half of the hypothalamus “roughly conforming to the suprachiasmatic and preoptic areas, is the site of a nervous structure which is of specific importance for the capacity of sleep”; he henceforth referred to this structure as a “sleep center”. Nauta also concluded via concurrent lesions to the rostral and caudal hypothalamus that active inhibition of the waking center(s) by the sleep center is how sleep is caused—his interpretation of the data was a prescient one and something we return to later in this review (see Section 5). A little over two decades later, in 1968, McGinty and Sterman reported that lesions of the basal forebrain (which included the tissue of the medially adjacent preoptic region) produced sleeplessness in cats, largely recapitulating Nauta’s findings [8].

## 2. Discovery of the Sleep-Active VLPO

A true watershed moment in the field of sleep science occurred when Saper and colleagues uncovered a bona fide ‘sleep center’ in the rostral hypothalamus, a previously unidentified “sleep-active” cell group in the ventrolateral preoptic area (VLPO) [9] of rats (See Table 1 for key references). The clearest density of these sleep-active VLPO neurons was located just lateral to the optic chiasm at the level of the crossing of the anterior commissure. Following the identification and anatomic characterization of the cellular VLPO, recordings made from other groups in the preoptic area, including presumptive VLPO neurons, revealed that many of these neurons fired more rapidly during sleep than during wakefulness, and more rapidly still when animals were recovering from sleep deprivation [10,11]. Some of the recorded VLPO neurons also showed a wake-active profile, although their role in sleep–wake regulation remains uncertain. These electrophysiological results correlated well with the finding that VLPO neurons accumulate cFos protein during sleep, but not during the accumulation of “sleep need” during prolonged wakefulness [9,12]. Subsequent single-unit recording studies found that VLPO neurons also start firing during sleep deprivation, a finding that was at some variance with the cFos studies [13]. To demonstrate that VLPO neurons were necessary to promote sleep, Lu and colleagues placed cell-specific lesions in the VLPO area in rats. Rats with verified bilateral VLPO lesions had a reduction of ~40–50% in total sleep time, along with sleep fragmentation; this was sustained for the post surgical survival period, which in some cases, was months [14]. These lesion findings were repeatedly replicated and have firmly established VLPO neurons as necessary for the maintenance of sleep [15,16,17,18]. However, animals with a complete, bilateral loss of VLPO still exhibit sleep. This is consistent with more recent studies that have identified and characterized other sleep-promoting systems in the brain, which can likely compensate, although clearly incompletely, following ablation of the VLPO. These sleep-promoting nodes include the GABAergic parafacial zone of the rostral medulla [19,20,21] and, most likely, the median preoptic nucleus (MnPO) [22] and are the focus of other reviews [23,24,25].

## 3. VLPO—Inputs and Outputs

In their initial paper, Saper and colleagues reported that sleep-active VLPO neurons provide heavy innervation of the tuberomammillary nucleus (TMN), an arousal-associated cell group in the caudal hypothalamus [9] (See Table 1 for key references). These authors subsequently showed that TMN-projecting VLPO neurons contain both GABA and the inhibitory peptide galanin, suggesting that the VLPO could exert potent inhibitory control over the TMN [26]. Gaus and colleagues later reported that most sleep-active VLPO neurons express mRNA for galanin in both nocturnal (mice and rats) and diurnal (degus) rodents as well as in cats [27]. On the basis of these findings, the authors hypothesized that GABA- and galanin-containing VLPO neurons might contribute to the sleep process through synaptic silencing of the brain’s arousal centers, which in many respects aligns with Von Economo’s original prediction [26]. Consistent with this theoretical model, it was later shown that VLPO galanin neurons provide innervation of most of the canonical components of the brain’s ascending arousal system, including the lateral hypothalamic orexin neurons, the serotonergic raphe neurons, the noradrenergic locus coeruleus and ventrolateral medullary neurons, and the glutamatergic parabrachial nucleus [28]. It had been previously reported that virtually all of these ‘arousal’ nodes provide reciprocal, and generally inhibitory, inputs to the VLPO [29]. The functional implications of these anatomic projections and connections are discussed below.

## 4. Cellular Anatomy of the VLPO

**Figure 1:**
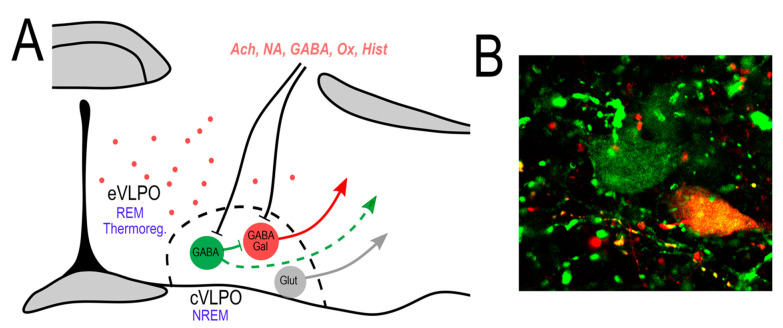
Cellular anatomy of the VLPO. (**A**) The VLPO core (cVLPO) contains several distinct neuronal populations including: (1) GABAergic neurons that express galanin (VLPO^Gal^), which are NREM sleep-promoting neurons; (2) GABAergic neurons that do not express galanin (VLPO^GABA^), which possibly suppress the activity of the VLPO^Gal^ neurons; and (3) glutamatergic neurons that presumptively promote arousal. There is also a subgroup of galanin neurons scattered in a region dorsal and medial to the core of VLPO, in the so-called extended VLPO (eVLPO). These neurons are involved in REM sleep and possibly in thermoregulation. Afferents to the cVLPO regulate the VLPO^Gal^ sleep-promoting neurons directly and/or indirectly through local VLPO^GABA^ neurons. These VLPO^GABA^ neurons might also project outside of the VLPO core. (**B**) Confocal image showing VLPO^Gal^ neurons (double labeled) and VLPO^GABA^ neurons (in green). In situ hybridization for Vgat in green and galanin in red.

The VLPO was initially described as a cluster of sleep-active neurons that co-express GABA and the inhibitory neuropeptide, galanin, and project to cell bodies and proximal dendrites of the TMN [9,27] (See Table 2 for Key References). Subsequent investigative work revealed the VLPO to be a brain region of even greater cellular heterogeneity, containing multiple neurochemically and functionally distinct cell populations. For instance, in addition to galanin, several other peptides (including peptides expected to excite their post synaptic targets) are produced and differentially expressed by VLPO neurons, including cholecystokinin (CCK), corticotropin-releasing hormone (CRH), dynorphin A and substance P [30,31], although the roles of most of these peptides in VLPO function remain unclear. While some degree of cellular overlap in VLPO peptide expressions has also been reported, e.g., some CCK- and substance P-expressing neurons also express CRH [30], the co-localization of peptide within VLPO cells does not appear to be a common feature of this nucleus. For a visual depiction of the cellular anatomy of the VLPO, discussed in more detail below, see Figure 1.

### 4.1. The GABAergic VLPO

The VLPO appears to contain a large number of GABA-releasing neurons, as indicated by the dense expression of the vesicular GABA transporter (Vgat). VLPO GABAergic neurons, however, comprise at least two distinct subgroups. The first expresses galanin (Gal; VLPO^Gal^), is sleep-active and is inhibited by wake-promoting inputs [13,27,32,33,34,35], while the second lacks galanin (VLPO^GABA^), is excited by arousal signals and might inhibit VLPO^Gal^ neurons to produce arousal [36,37]. The latter and more recently ‘discovered’ second subgroup of VLPO^GABA^ neurons has received considerable attention as of late and is discussed in detail below.

### 4.2. The Galaninergic VLPO

Galanin is an inhibitory peptide, and upon its co-release with GABA, it is likely to contribute to the inhibition of wake-promoting neurons [38,39]. Supporting this concept are results from in vitro electrophysiology studies showing that the locus coeruleus (LC), TMN, dorsal raphe nucleus (DRN) and orexin neurons are all inhibited by galanin [39,40,41,42] and that all these regions are innervated by VLPO [26,28,43]. Nevertheless, the relative contribution of GABA versus galanin signaling to promoting NREM sleep remains unclear. Answering this question will likely require an intersectional genetic strategy to delete either the Vgat or the galanin genes selectively in galanin-expressing VLPO neurons, but to our knowledge, these tools are not currently available. Regarding whether or not the synaptic release of galanin by VLPO neurons is required to produce sleep, there remains a general consensus that galanin is the best available cellular ‘marker’ within the VLPO for identifying sleep-promoting neurons, and this is true across multiple species [27]. Further indications of strong phylogenetic conservation in the role of this cell group in sleep–wake regulation are found in post mortem studies in humans, which established a close correlation between the number of galanin-immunoreactive neurons in the intermediate nucleus of the hypothalamus (or human homolog of the VLPO) and the amount/percentage of consolidated sleep in aged individuals [44,45].

It is also worth noting that there appear to be at least two functionally distinct galanin-expressing populations in the VLPO region. The first of these subgroups is found within the anatomic boundaries of the VLPO “core” or “cluster” (cVLPO^Gal^) whereas the second subgroup is located in a dorsal and medial position to the core in the so-called “extended” VLPO (eVLPO^Gal^) [14,27,46]. From a functional standpoint, cVLPO^Gal^ neurons are thought to promote NREM sleep, whereas eVLPO^Gal^ neurons are more strongly linked to the regulation of REM sleep [14,46]. With respect to the latter, the idea of the role of eVLPO^Gal^ neurons in REM sleep regulation increasingly gained support. For example, single-unit recording studies demonstrated that the eVLPO region contains REM-active neurons [13,47,48,49], and tracing studies have shown that the eVLPO^Gal^ neurons innervate key pontine nuclei implicated in REM sleep regulation [46,50,51]. However, the lack of a selective marker for distinguishing between cVLPO^Gal^ and eVLPO^Gal^ neurons and the fact the cVLPO and the eVLPO are very small nuclei that are located in close proximity to one another, have proven a challenge for researchers seeking to “parse” the respective roles of these anatomically distinct nuclei in behavioral state control.

### 4.3. The Glutamatergic VLPO

In addition to GABA-releasing neurons, the VLPO also contains glutamate-releasing neurons, as indicated by the expression of the vesicular glutamate transporter 2 (Vglut2) (Allen Brain Atlas, n.d.). Interestingly, the activation of glutamatergic VLPO (VLPO^Vglut2)^ neurons suppresses sleep, delays REM sleep and consolidates wakefulness for about an hour [52]. The optogenetic stimulation of glutamatergic neurons in the preoptic area, including the VLPO, also produces rapid transitions from NREM to wakefulness [30]. Taken together, these findings suggest that VLPO^Vglut2^ neurons, upon their activation, function to promote waking rather than sleep. The circuit bases by which glutamatergic VLPO neurons contribute to arousal remains unresolved, although there are data to suggest that VLPO glutamatergic neurons might promote wakefulness by activating other arousal centers, such as the TMN [30]. Regardless, the arousal response elicited by the acute activation of VLPO glutamatergic neurons is relatively short in duration when compared to the responses evoked by the comparable activation of other wake-promoting neurons in the brain, which in some cases, consistently produce sustained wakefulness for three to six hours, even when this activation occurs during a period of high sleep drive [53,54,55,56].

## 5. VLPO Regulation of Behavioral State: Circuit Basis of Sleep and Arousal

How the brain transitions between the behavioral states of wake and sleep (and REM sleep) remains incompletely understood. One highly influential circuit model for state transitions, termed the “flip-flop” switch model, posits reciprocal inhibitory interactions between sleep- and wake-promoting cell groups, similar to the function of an electronic “flip-flop” switch/circuit [25,57,58]. Within the framework of the flip-flop model, the VLPO represents the “sleep side”, whereas the ascending arousal nodes represent the “arousal side”. That these cell groups exhibit, respectively, sleep- and wake-active firing profiles has provided general support for the model. More specifically, in this flip-flop design, the VLPO inhibits the arousal nodes (e.g., TMN, LC and DR neurons) through the synaptic release of GABA and galanin, whereas the TMN, LC and DR inhibit the VLPO through the synaptic release of histamine, noradrenaline and serotonin, respectively [59,60]. By virtue of the self-reinforcing nature of these switches—that is, when each side is firing they reduce their own inhibitory feedback—the flip-flop switch is inherently stable in either end state, but avoids intermediate states. Hence, the flip-flop design ensures the stability of behavioral states, but also facilitates relatively rapid switching between behavioral states. One design flaw of flip-flop switches is that the frequency of unwanted state transitions can increase if one side of the switch is “weakened”, as the weakened side becomes less able to inhibit the other side, thereby biasing the switch toward a midpoint where smaller perturbations may trigger a state transition. As an example, cell loss in the VLPO, which occurs during aging, may weaken the “sleep side” of the switch, which would predictably lead to sleep fragmentation and daytime napping, both of which are frequent complaints in the elderly [44]. While many features of the theoretical framework of the flip-flop model were discovered experimentally, other features have proven more difficult to convincingly demonstrate. For example, the LC and DRN are directly inhibited by the VLPO through the release of GABA, and the LC and DRN directly inhibit VLPO sleep-active neurons through the release of noradrenaline and serotonin, whereas reciprocal inhibition between the VLPO and the TMN necessarily requires additional synaptic relays; this is discussed in more detail below. Specifically, while the VLPO directly inhibits TMN neurons through the release of GABA, histamine released from the TMN does not inhibit the VLPO directly, but rather activates GABAergic interneurons that, in turn, inhibit the VLPO, i.e., feedforward inhibition [36,61]. Hence, while reciprocal inhibition per the flip-flop model is conserved, it does require further circuit elements, which in this example takes the form of GABAergic interneurons. Additionally, and as previously indicated, behavioral state transitions are not always as rapid as would be predicted by the flip-flop model, instead they often occur gradually, i.e., over minutes rather than seconds.

Rather strikingly, given the influential nature of this model and with the exception of a recent paper showing that the noradrenergic LC promotes arousal by inhibiting the activity of the VLPO [37], there exists little in vivo evidence that monoaminergic or cholinergic inhibition of the VLPO promotes EEG or behavioral arousal. Conversely, the flip-flop model does not fully consider the possibility that non-aminergic and non-cholinergic cell groups that innervate the VLPO may also contribute to sleep–wake regulation. Inspired by this knowledge gap, our labs recently explored this question and found that wake-promoting (and wake-active) GABAergic neurons of the lateral hypothalamus (LH^GABA^) [54], when acutely activated, can rapidly produce arousal from deep sleep by inhibiting sleep-promoting galanin neurons of the VLPO [62]. These results were the first to identify a long-range, non-aminergic and non-cholinergic inhibitory input that can effectively “turn off” the sleep-promoting neurons of the VLPO to promote arousal in vivo. We also found that using conditional retrograde tracing, which the LH^GABA^ neurons themselves receive as a prominent input from the VLPO, supported the hypothesis that sleep-promoting VLPO and wake-promoting LH^GABA^ neurons could exist in a state of mutual inhibition. Therefore, LH^GABA^ neurons may comprise an additional, non-aminergic and non-cholinergic circuit component of the flip-flop switch.

As indicated, our understanding of how VLPO^Gal^ neurons are regulated, both by long-range monoaminergic and cholinergic inputs as well as by local (inter)neurons, is relatively immature. We know for example that VLPO^Gal^ neurons are inhibited by monoamines [33,34,59,63] and that monoaminergic inputs to the VLPO originate from the LC and the C1/A1 adrenergic cell group of the ventrolateral medulla (VLM) [29]. Accordingly, electrical and optogenetic stimulation of the LC or of the VLM inhibits the firing of sleep-active neurons in VLPO via α2-mediated effects [37,64], and promotes cortical activation, presumably as a direct function of inhibiting sleep-active VLPO neurons. VLPO^Gal^ neurons are similarly strongly inhibited by cholinergic agonists [59,65], although the source of cholinergic input(s) to the VLPO remains unclear, in particular as retrograde tracing studies determined that cholinergic inputs to the VLPO are rare, if nonexistent [29]. The apparent mismatch between cholinergic innervation and cholinergic receptors in the VLPO area remains a mystery.

The VLPO also receives inputs from other canonical arousal-related nodes, including lateral hypothalamic orexin neurons and, as indicated, the histaminergic TMN [29]. Targeted infusion of orexin or histamine into the VLPO produces rapid arousal responses [36,66,67]. Interestingly, during wakefulness, orexin and histamine inhibit VLPO^Gal^ neurons, although they do not do so directly. Their arousal responses appear to be produced through an indirect feedforward GABAergic mechanism [36,61,68]. The source of increased GABAergic tone providing inhibitory control over VLPO^Gal^ neurons during arousal, which can potentially promote arousal, remains unresolved. Orexin and histamine could, for instance, activate terminals of GABAergic afferences to the VLPO, or they could activate collaterals from local (i.e., intra-VLPO) GABAergic neurons. The available data support the latter model [36,68].

The results from recent studies have further suggested that, similar to orexin and histamine, other arousal signals may also inhibit VLPO^Gal^ neurons through an internal GABAergic circuit. For instance, noradrenaline and carbachol inhibit VLPO^Gal^ neurons directly [33,34,59] and indirectly through increasing GABA release [37]. Noradrenaline and carbachol directly inhibit the VLPO^Gal^ neurons via α2 adrenergic and muscarinic receptors [37,48,65,69], respectively, and noradrenaline can further increase GABAergic-afferent input via α1 adrenergic receptors, indicating an additional and synergistic mechanism of inhibition [37].

In summary, the VLPO is regulated by afferent inputs from many sources [29]. While some of these inputs provide a direct synaptic regulation of the VLPO^Gal^ sleep-promoting population, some afferences also appear to operate through a local (i.e., intra-VLPO) GABAergic circuit. The available data support the hypothesis that local GABAergic neurons within the VLPO (VLPO^GABA^), which provide collaterals to VLPO^Gal^ neurons, function as an interface between afferent inputs to the VLPO and VLPO^Gal^ neurons, effectively serving as a common ‘point of entry’ through which wake and sleep signals control VLPO^Gal^ neurons to regulate arousal, e.g., wake-promoting signals (via feedforward inhibition) inhibit VLPO^Gal^ neurons to produce arousal- and sleep-promoting signals (via disinhibition), activating VLPO^Gal^ neurons to produce NREM sleep. This local GABAergic mechanism for promoting state transitions could be shared by most, if not all, wake- and sleep-promoting inputs that modulate VLPO activity. According to this model, VLPO^GABA^ neurons are wake-active and possibly wake-promoting. Recordings in the VLPO have uncovered neurons that are active in wakefulness [13]. Additionally, unlike the activation of VLPO^Gal^ neurons, the optogenetic activation of GAD2-expressing VLPO neurons (which likely activates both VLPO^Gal^ and VLPO^GABA^ neurons) produces arousal, suggesting that the VLPO contains wake-promoting GABAergic neurons that can override the co-activation of sleep-promoting VLPO^Gal^ neurons [30].

### Sleep Promoting Signals in VLPO—The Role of Adenosine and Prostaglandin D2

Adenosine (AD) and prostaglandin D2 (PGD_2_) are arguably the most widely recognized and intensively investigated endogenous sleep-promoting signals [70,71,72] (See Table 1 for key references). They also appear to be inextricably linked to the mechanism by which they promote sleep. Both AD and PGD_2_ progressively accumulate in the extracellular space during wakefulness and their levels continue to rise during sleep deprivation, [73,74] eventually driving sleep by inhibiting wake-promoting neurons throughout the brain and/or disinhibiting VLPO neurons. In addition, PGD_2_ levels are elevated during inflammation and are thought to mediate, at least in part, the sleepiness associated with infections and inflammation. For example, PGD_2_ levels are elevated in the CSF of patients with African sleeping sickness, as well as in the serum of narcoleptic patients, the levels in the latter correlating with excessive daytime sleepiness but not cataplexy [75,76].

AD itself is a purine metabolite that has long been linked with the sleep process [77]. For example, the subarachnoid administration of AD agonists promotes sleep and induces the expression of the cFos protein in VLPO neurons [78]. AD appears to exert these effects, at least in part, via adenosine A1 receptors to disinhibit VLPO^Gal^ neurons, i.e., by reducing GABAergic synaptic inputs on sleep-promoting VLPO neurons [79,80]. Pharmacological studies also suggested that AD may directly activate A2a receptors [33], although to the authors’ knowledge, it is not yet been determined if the A2a receptor is expressed in VLPO neurons, VLPO^Gal^ or otherwise.

Prostaglandins (PG), on the other hand, are a group of lipid compounds also known as eicosanoids. The specific role of PGD_2_—which is the most abundant PG in the brains of rats [81] and other mammals, including humans [82]—in sleep regulation has been identified. Initial and seminal research on PGD_2_ in sleep regulation was performed by Uneuo and colleagues [83,84,85,86], who reported that infusion of PGD_2_ into the lateral ventricles or into the subarachnoid space at the base of the forebrain, but not other regions of brain parenchyma, could induce sleep. Similar hypnogenic effects of ICV infusion of PGD_2_ were subsequently demonstrated in primates [87]. Importantly, animals under PGD_2_-induced sleep remain arousable, and the EEG power spectrum of NREM sleep is the same as that of natural sleep [71]. The concentration of PGD_2_ in the CSF also shows a strong diurnal rhythm that oscillates in parallel with the sleep–wake cycle [88], and notably, the amplitude of this oscillation increases during sleep deprivation, i.e., with an increase in sleep propensity [74]. Within the brain, PGD_2_ is produced in the leptomeninges, choroid plexus, and oligodendrocytes [89]. The main cognate receptor for PGD_2_ is the DP1 receptor, which is primarily expressed in the leptomeninges of the ventral surface of the rostral basal forebrain of mice. These DP1-enriched leptomeninges were immunohistochemically defined as bilateral wings under the rostral basal forebrain lateral to the optic chiasm, and thus were in close proximity to the anterior preoptic hypothalamus region containing the sleep-promoting VLPO neurons [86]. In fact, administration of PGD_2_ into the subarachnoid space at the base of the rat forebrain produces sustained NREM sleep (with minimal effects on REM sleep), and this sleep-inducing effect is absent in DP1-KO mice [86], indicating that NREM sleep induction by PGD_2_ is mediated by the DP1 receptors. However, in DP1-KO mice, it was further observed that their baseline sleep–wake patterns were essentially identical to WT mice, suggesting that DP1 receptors may not be crucial for basal sleep–wake regulation, or that the lack of receptors in these mice was compensated, developmentally or otherwise [86]. Yet, DP1-KO mice also exhibited a strongly attenuated rebound of NREM sleep following sleep deprivation, suggesting that endogenous PGD_2_ might be involved in the homeostatic regulation of NREM sleep [71,90]. Additionally, while the cellular bases of these effects are incompletely understood, it was shown that the administration of PGD_2_ into the subarachnoid space markedly increases the expression of cFos in VLPO neurons and the basal forebrain leptomeninges [91]. Given that the DP1 receptors are not expressed in the brain parenchyma, but rather only in the leptomeninges [86,92], these results suggest that PGD_2_ may act via the leptomeninges to activate VLPO neurons, and thereby promote sleep. Of additional interest, PGD_2_ infusion into the subarachnoid space of mice increases extracellular adenosine concentrations in a dose-dependent manner [86], and this effect is absent in DP1-KO mice [86].

Therefore, in summary, one current model that remains to be critically tested is the idea that PGD_2_ acts via DP1 receptors in the leptomeninges to induce the production of AD. This then activates sleep-promoting VLPO^Gal^ neurons, and AD most likely carries out this process by acting in the A1 receptors (i.e., indirectly through disinhibition) but could possibly act via the direct activation of A2a receptors [78,85].

## 6. VLPO and Other Physiological Processes

### 6.1. VLPO and Thermoregulation

Recent work has suggested that the VLPO, or more likely a subset of VLPO neurons, may contribute to thermoregulatory processes (See Table 1 for key references). For example, in addition to triggering sleep, the selective chemoactivation of VLPO^Gal^ neurons produces a marked hypothermia [28]. This finding is perhaps unsurprising given that a rapid decrease in core temperature is known to occur upon the onset of natural sleep [93,94,95], and that the preoptic region, likely including the VLPO, contains heat-sensitive neurons (i.e., neurons that increase their firing rates in response to local temperature increases) [49,96,97]. It has also been hypothesized that the activation of heat-sensitive preoptic neurons by increased core temperature may also subserve the connection between fever and sleepiness [98,99]. Regardless, the reduction in core temperature that occurs upon sleep onset is largely due to skin vasodilation, i.e., heat loss [100], and this process is linked with the inhibition of thermoregulatory neurons in the dorsomedial hypothalamic nucleus (DMH) and the raphe pallidus (RPa) [101,102]. However, it is the authors’ surmise that the promotion of NREM sleep and hypothermia are controlled by two distinct, but partially intermixed, VLPO^Gal^ cell populations, with those generating NREM sleep mainly located in the cVLPO, and those inducing heat loss mainly located in the dorsal and medial eVLPO [28,103]. In general support of this concept, we note from the authors’ heatmaps in the chemogenetic study of VLPO^Gal^ neurons ([28], Figure 4) that the simultaneous or otherwise joint activation of cVLPO^Gal^ and eVLPO^Gal^ neurons was near-certain, and thus would trigger both NREM sleep and hypothermia. Furthermore, and in the same study, optogenetic activation, which permitted a more selective targeting of cVLPO^Gal^ neurons (i.e., with fiber optics placed just above the cVLPO) promoted NREM sleep, but not hypothermia [28], whereas the optogenetic stimulation of GABA neurons located more dorsally, in an area roughly corresponding to the eVLPO, produces hypothermia [103]. Finally, tracing and physiological studies have shown that the region dorsal and medial to the cVLPO provides an inhibitory innervation of the DMH and the RPa and that the activation of this circuit can trigger heat loss through arterial vasodilation in the tail [104,105,106]. Of interest, more recent data suggested that neurons within the cVLPO may also contribute to this pathway, but that the functional cell group involved is most likely the (putative wake-promoting) VLPO^Vglut2^ and not the sleep-promoting cVLPO^Gal^ neurons [107].

### 6.2. VLPO as Metabolic Sensor

An association between sleep and satiety is long-established, albeit largely anecdotally. Known as post prandial somnolence, aka “food comas”, this phenomenon has historically been linked with changes in post meal insulin (or other metabolic factors) or the activation of ascending gastrointestinal (vagal) inputs to the brainstem, although neither of these theories have been rigorously tested. However, recent studies suggested that sleep-active VLPO neurons might be capable of directly sensing metabolic changes, and thus might contribute to the sleep or sleepiness that accompanies a large meal. The infusion of glucose into the VLPO also promotes NREM sleep and increases cFos expression [108]. Interestingly, glucose-driven cFos activation in the VLPO appears to be a direct response to glucose. Glucose within the physiological range selectively excites putative VLPO sleep-promoting neurons [108] via the same cellular mechanism that occurs in glucose-sensing pancreatic beta cells and POMC neurons in the arcuate nucleus [109,110]. Specifically, glucose is initially transported intracellularly and then catabolized via glycolysis, which leads to an increase in ATP levels and induces the closure of K_ATP_ channels and membrane depolarization [111]. Satiety signals may therefore promote sleep by activating sleep-active VLPO neurons, which is an interesting parallel phenomenon to that of the hunger- and hypoglycemia-driven activation of centers, which drive arousal to facilitate foraging [112]. Therefore, while wakefulness/alertness is enhanced with fasting by the activation of wake-promoting neurons, resting (i.e., sleeping/napping) is favored after food intake and direct activation of VLPO sleep-promoting neurons may contribute to this change in behavioral state [113,114,115,116].

### 6.3. VLPO and Anesthesia

Over the past two decades, there has been an increased interest in the relationship between sleep and general anesthesia [117,118]. One emerging hypothesis is that loss of consciousness in anesthesia might rely on the same circuitry that produces natural sleep, in particular for anesthetics that may interact with and activate sleep-promoting neurons in the preoptic area, including the VLPO. This hypothesis derives support from the finding that putative sleep-promoting neurons in the VLPO are directly activated by volatile anesthetics, such as isoflurane and halothane, and that general anesthesia induces cFos expression in VLPO neurons [63,69,119,120,121]. However, both VLPO lesion studies and the selective activation of GABAergic or glutamatergic neurons in VLPO have found an absence of effect of these manipulations on anesthetic induction or recovery time [16,52]. Interestingly, sleep deprivation increases sensitivity to anesthesia and prolongs time to emergence [122]. Therefore, sleep loss could explain why, paradoxically, the extent of cell loss following lesions of the VLPO correlates with deeper, rather than lighter, anesthesia and delayed, rather than quicker, emergence [16]. Hence, while sleep-promoting neurons in the VLPO are activated during anesthesia, their activation is unlikely to be necessary for the loss of consciousness induced by general anesthesia.

## 7. On the Horizon for VLPO Research

While a subjective assessment, the authors of this review have identified several current and important knowledge gaps regarding the neurobiology of the VLPO. The first of these gaps is the incompletely understood role that putative wake-promoting glutamatergic VLPO neurons play in the regulation of behavioral state (Figure 1). More specifically, we think it will be important to identify and characterize their afferent inputs and efferent outputs and to determine whether, in addition to glutamate, they express and co-release any of the neuropeptides found in the VLPO region. Another important knowledge gap is how transcriptional profiles may vary across VLPO cell subtypes. For example, do VLPO^GABA^ neurons, which appear to function as a critical interface between VLPO^Gal^ neurons and modulatory afferent inputs, express unique GPCRs? If so, these could form the basis for newer and more targeted pharmacologic approaches to treating the crippling inability to maintain consolidated wakefulness in a host of neurodegenerative, neurodevelopmental, and neuropsychiatric diseases, in which disordered arousal, including even hyperarousal (e.g., insomnia), is a prominent clinical feature. The same question can be asked about the cVLPO^Gal^ and eVLPO^Gal^ cell populations, which themselves may be transcriptionally heterogeneous, and thus further subdivided into functionally distinct cell clusters.

## 8. Summary

Without doubt, challenges lie ahead as we seek a more refined understanding of the neurobiology of the VLPO, but the ever-expanding toolkit of newer methods and technologies will unquestionably facilitate deeper and more detailed interrogations of the cellular and molecular VLPO. It is our expectation that, within the next decade or two, experimental work will reveal druggable targets in key VLPO subpopulations that will in turn permit the development of newer drugs that can be clinically deployed for treating a wide range of sleep- and arousal-based disorders without side effects. Our confidence in this outcome is based on the fact that in the span of just 25 years, the field has gone from being unaware that the VLPO was a bona fide *schlafsteuerungszentrum*, to our current and impressively detailed understanding of the VLPO’s heterogeneous cellular composition, how these cells are modulated by pre-synaptic inputs, and a far more granular understanding of their intra-nucleus circuitry.

**Table 1:** Selected key references.

**The discovery of VLPO as sleep-promoting nucleus**	The VLPO contains sleep-active neurons [9,11,13]; VLPO sleep-active neurons express GABA and galanin [22,26,27]; VLPO neurons are inhibited by wake-promoting signals [59]; VLPO inhibits wake-promoting neurons (see [26,28] and, for reviews, [123,124]); Lesions of the VLPO produce insomnia [14]; Activation of VLPO galanin neurons promotes NREM sleep [28].
**Afferent and efferent inputs and cellular composition**	VLPO afferent [29] and efferent [26,28,51,125] inputs; Cellular composition [22,26,27,30,31,52].
**Electrophysiology and pharmacological studies**	In vitro electrophysiology studies Wake signals inhibit VLPO neurons directly [33,34,59,63] and indirectly via GABA transmission [35,36,61,68]; Adenosine activates VLPO neurons [33,79,80]; Glucose activates VLPO neurons [108,126]; Volatile anesthetics activate VLPO neurons [63,69,121]. Whole-animal pharmacological studies Infusion of wake-promoting signals into VLPO promotes arousal [35,36,66,67]; Adenosine and PGD_2_ are sleep-promoting signals, and their infusion into the subarachnoid space activates VLPO neurons [78,79,83,86,91].
**Regulation of behavioral states**	Lesions of the VLPO produce insomnia [14,15,16,17,127,128]; Chemogenetic and optogenetic studies [28,30]; Flip-flop model (see [54,61,62] and, for reviews, [25,123]); VLPO and REM sleep [13,14,46,47,48,49].
**Other physiological processes**	Thermoregulation: Heat-sensitive neurons in the VLPO region [49,96,97,129]; Chemogenetic and optogenetic studies [28,103]; VLPO projections to thermoregulatory nuclei [28,106,107]; Suggested reviews on thermoregulation sleep and preoptic area [99,130,131,132,133]. Metabolic Sensor: Glucose infusion into the VLPO region promotes NREM sleep [108]. Anesthesia: Activation of VLPO neurons [63,69,119,120]; Manipulation(s) of VLPO neurons alter anesthesia [63,69,134] or have no effect on anesthesia [16,52].

**Table 2:** Cellular anatomy of the VLPO: selected key references.

**The VLPO nucleus**	The VLPO was initially defined as a cluster of sleep-active GABAergic and galaninergic neurons that project to the TMN [9,27]; The VLPO contains multiple cell populations of GABAergic and glutamatergic neurons, many of which co-express peptides, including galanin, CCK, CRH, dynorphin A and substance P [27,30,31].
**Galaninergic neurons**	Galanin is the best available marker for identifying sleep-promoting VLPO neurons [27,28]; VLPO galaninergic neurons express GAD and Vgat, indicating that they are GABAergic and release synaptic GABA [26,30]; VLPO galaninergic neurons are inhibited by wake-promoting signals [33,34,36,59]; Activation of galaninergic neurons in the core of the VLPO (cVLPO^Gal^) promotes NREM sleep [28]; Activation of galaninergic neurons in the extended VLPO (eVLPO^Gal^) promotes REM sleep [14,46] and possibly hypothermia [28].
**GABAergic neurons**	In the VLPO, there are at least two subgroups of GABAergic neurons [36,59]; The first group expresses galanin (VLPO^Gal^), is sleep-active and NREM sleep-promoting [26,28]. These neurons are also inhibited by wake-promoting signals [33,34,36,59]; The second group lacks galanin (VLPO^GABA^), is excited by arousal signals, is wake-active and promotes arousal [30,36,37].
**Glutamatergic neurons**	VLPO-glutamatergic neurons promote arousal [30,52].

## Data Availability

Not applicable.
